# Supplementary material for: Evaluation of in situ tissue-engineered arteriovenous grafts suitable for cannulation in a large animal model
Source: Commun Mater. 2025 Jul 16;6(1):151. doi: 10.1038/s43246-025-00879-z (PMC12267051; doi:10.1038/s43246-025-00879-z)
Supplement: Supplementary file 2 — Supplemental Material [file 43246_2025_879_MOESM2_ESM.pdf]

Evaluation of In Situ Tissue-Engineered Arteriovenous Grafts Suitable for Cannulation in a Large Animal Model

Paul J. Besseling[1,2], Wojciech Szymczyk[3], Martin Teraa[1,2], Raechel J. Toorop[2], Paul. A.A. Bartels[3], Boris Arts[3], Rob C. H. Driessen[3], Arturo M Lichauco[3], Hidde C. Bakker[1], Joost O. Fledderus[1], Gert J. de Borst[2], Patricia Y.W. Dankers[3], Carlijn V.C. Bouten[3], Marianne C. Verhaar[1].

1: Department of Nephrology and Hypertension, Regenerative Medicine Centre, University Medical Centre Utrecht, Utrecht, the Netherlands
2: Department of Vascular Surgery, University Medical Centre Utrecht, Utrecht, the Netherlands
3: Department of Biomedical Engineering, and Institute for Complex Molecular Systems, Eindhoven University of Technology, Eindhoven, the Netherlands

Running Title:
Cannulation of Tissue Engineered AV-graft

Corresponding author:
Marianne C. Verhaar
Dept. of Nephrology and Hypertension
M.C.Verhaar@UMCUtrecht.nl

Addresses:
University Medical Centre Utrecht
P.O. Box 8599
3508 GA Utrecht
The Netherlands
Tel +31 88 75 573 29

Eindhoven University of Technology
PO Box 513
5600 MB Eindhoven
The Netherlands
+31 40 247 9111

Keywords:
AV shunt
In Situ tissue engineering
Vascular dialysis access
Repeated cannulation
Biodegradable supramolecular material
Goat model

Supplemental Figures & Tables

Table S1 - Implantation Set-up

	Left	Cannulation	Right	Cannulation	Explant
Goat #1	Sham		TE	Yes	12 weeks
Goat #2	TE	Yes	ePFTE	Yes	12 weeks
Goat #3	TE	No	TE	Yes	4 weeks
Goat #4	ePTFE	Yes	TE	Yes	4 weeks
Goat #5	TE	Yes	Sham		12 weeks
Goat #6	TE	Yes	ePTFE	Yes	12 weeks
Goat #7	TE	No	TE	Yes	12 weeks
Goat #8	TE	Yes	TE	No	12 weeks

TE: Tissue engineered graft; **Sham**: Clamping only, no implantation; **ePTFE**: expanded-polytetrafluoretheen

Table S2 - qPCR primer list

Target		*Sequence (5'--> 3')*	*Tm*	*Length*	*Ampl. Size*
**B2M**	F	TGTCCCACGCTGAGTTCACT	60	24	137
	R	TGAGGCATCGTCAGACCTTGA		20	
**18S**	F	ATGCAGAATCCACGCCAATAC	59	21	147
	R	GGCCCGAATCTTCTTCAGG		19	
**Collagen I**	F	CCCTTCTTGGTCAGACTCCC	59	20	166
	R	GGCTGGCTAGAGGGGATAAA		20	
**Collagen III**	F	AACGGCATCAAAGGACATCG	59	20	158
	R	CCACTTGTTCCATCCTTGCC		20	
**aSMA**	F	TCATGAGCAGAGCAGTGGAA	59	20	209
	R	GGTGGTGTTCTGGAAAGCTG		20	

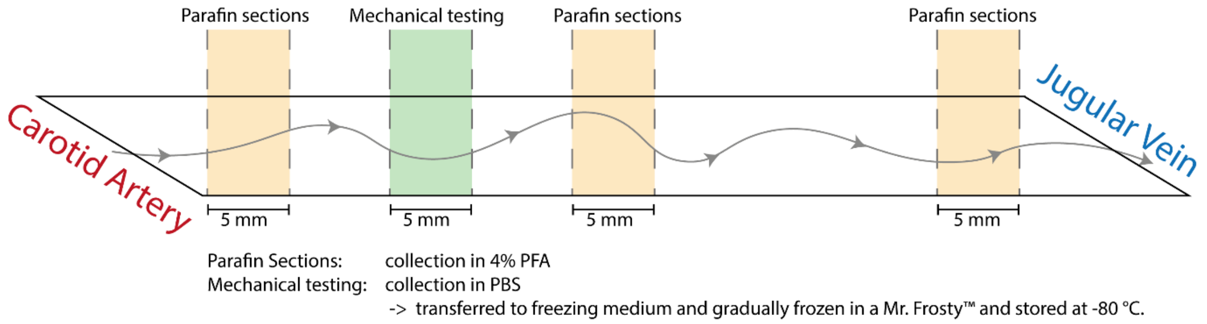

Figure S1. Cutting scheme of graft explants. 5 mm samples are taken form the aortic side, middle and jugular side of the graft in 4% PFA for paraffin sectioning. Additionally, a sample for mechanical testing and SEM is collected in cold PBS and transferred to freezing medium and gradually frozen for storage. The remaining graft is snap frozen for future analysis.

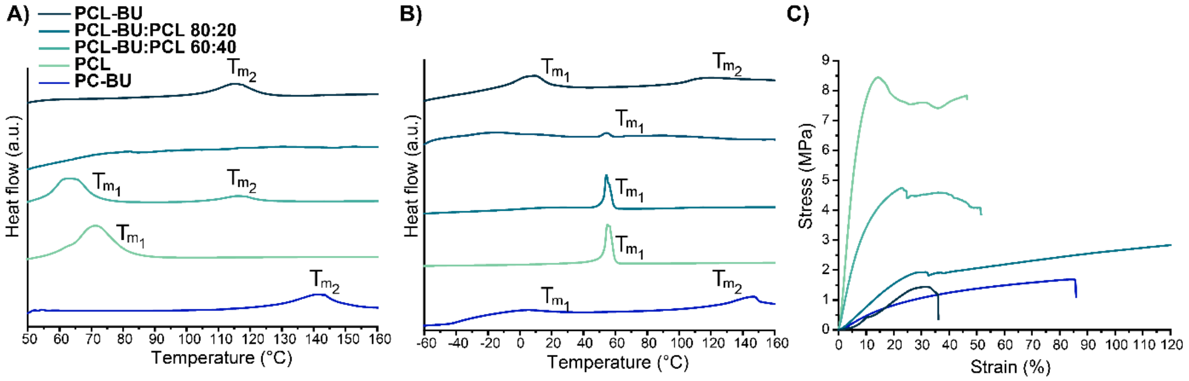

	Young's Modulus (MPa)	First heating run				Second heating run				
		T_{m1} (°C)	ΔH_1 (J/g)	T_{m2} (°C)	ΔH_2 (J/g)	T_g (°C)	T_{m1} (°C)	ΔH_1 (J/g)	T_{m2} (°C)	ΔH_2 (J/g)
PCL-BU	7.9 ± 0.09	-	-	115.1	13.0	-59.5	9.2	16.3	117.5	10.0
PCL-BU:PCL 80:20	8.1 ± 0.06	-	-	-	-	-63.1	54.3	5.2	-	-
PCL-BU:PCL 60:40	42.1 ± 0.4	63.1	28.4	116.1	6.9	-60.0	52.5	23.7	-	-
PCL	107.4 ± 0.8	70.0	79.4	-	-	-62.1	56.4	67.2	-	-
PC-BU	5.5 ± 0.03	-	-	141.3	16.8	-49.8	-1.5	15.6	146.4	9.6

Figure S2. Thermal and mechanical properties of 3D-printed flat coil samples consisting of PC-BU, PCL-BU, PCL, or a combination of PCL-BU: PCL. Thermograms showing the first (left) and second (right) heating runs (30 °C/min and 10 °C/min respectively) as measured with DSC with the peaks being endothermic processes **(A, B).** Stress-strain curve of the different coils **(C)**. Table with moduli determined from the stress-strain curves as well as the melting peaks with corresponding enthalpy change calculated from the first and second heating runs of the DSC thermograms **(D)**.

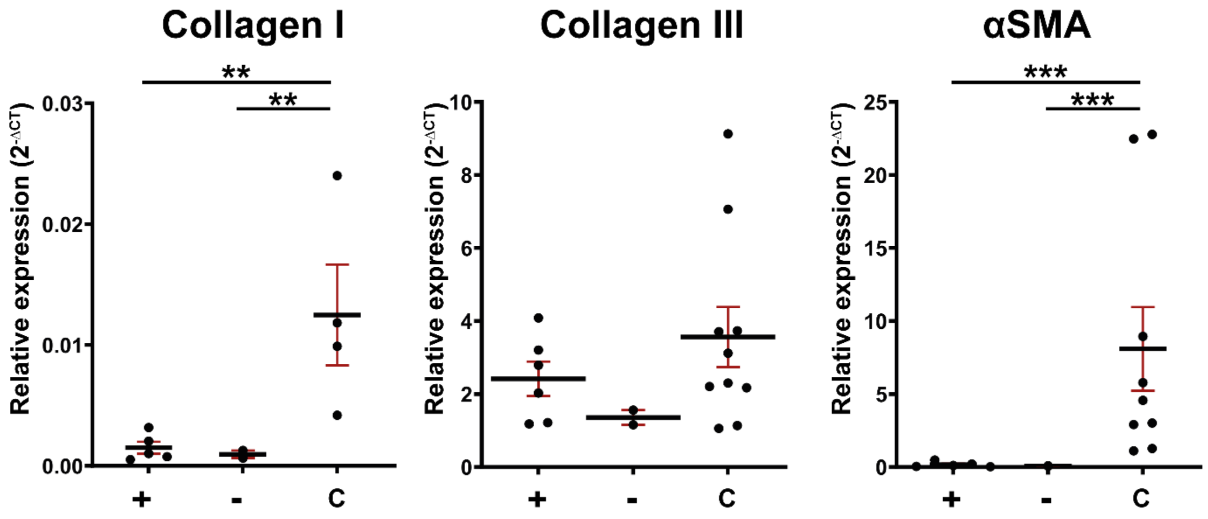

Figure S3. – Relative gene expression (2Δ^{-CT}) of Collagen I, Collagen III and αSMA compared between cannulated(*n* = 6),, non- cannulated grafts (n=2) grafts at 12 weeks and native carotids (*n* = 10) as controls. One-way ANOVA with Tukey's post-hoc

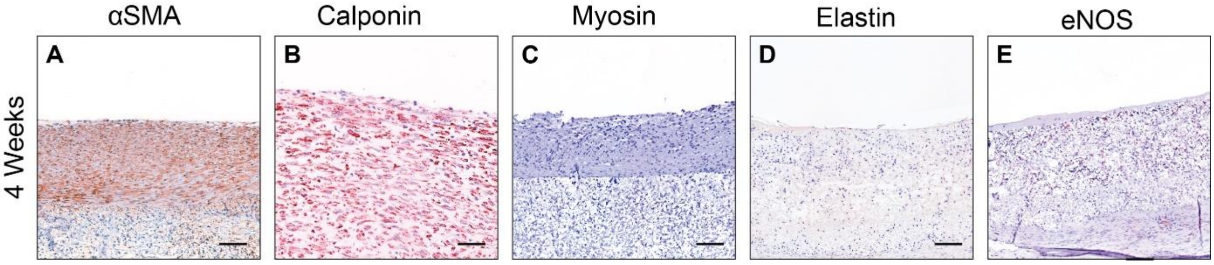

Figure S4. – Immunohistochemistry on vascular markers. Representative transverse sections of the center part of the grafts, (non)-Cannulated explanted at 4 weeks. Stained with Novared® for smooth muscle marker αSMA **(A),** early contractile marker Calponin **(B),** mature contractile marker Myosin Heavy Chain **(C),** Elastin **(D),** and endothelial marker eNOS **(E).** 1 month (1M), Non-cannulated (-), Cannulated (+), Carotid Artery (C). Scale bars 50µm. Data are shown as mean±SEM. *p <0.05, **p <0.01.
