# Supplementary material for: Evaluation of in situ tissue-engineered arteriovenous grafts suitable for cannulation in a large animal model
Source: Commun Mater. 2025 Jul 16;6(1):151. doi: 10.1038/s43246-025-00879-z (PMC12267051; doi:10.1038/s43246-025-00879-z)
Supplement: Supplementary file 3 — Description of Additional Supplementary File [file 43246_2025_879_MOESM3_ESM.pdf]

Description of additional supplementary file

File name: Supplementary movie 1

Description: Mechanical testing

File name: Supplementary movie 2

Description: Cannulation
